# Effective spectrum-based antibiotic resistance index for monitoring resistance in Gram-negative bacilli

**DOI:** 10.1017/ash.2025.10275

**Published:** 2026-03-27

**Authors:** M. Cristina Vazquez Guillamet, Alice Bewley, Nicole J. Tarlton, Reid Goodman, Michael J. Durkin, Michael Bernauer, Meghan Brett, Kevin Hsueh, Cristian Bologa, George Turabelidze, Andrew Atkinson, Victoria J. Fraser

**Affiliations:** 1 Division of Infectious Diseases, Department of Medicine, Washington University, St. Louis, MO, USA; 2 Division of Pulmonary and Critical Care Medicine, Department of Medicine, Washington University, St. Louis, MO, USA; 3 Department of Pathology and Laboratory Medicine, Endeavor Health, Evanston, IL, USA; 4 College of Pharmacy, University of New Mexico, Albuquerque, NM, USA; 5 Division of Infectious Diseases, Department of Internal Medicine, University of New Mexico, Albuquerque, NM, USA; 6 Division of Translational Informatics, Department of Internal Medicine, University of New Mexico, Albuquerque, NM, USA; 7 Missouri Department of Health and Senior Services, St. Louis, MO, USA; 8 Department of Medicine, Washington University, St. Louis, MO, USA

## Abstract

**Background:**

Antimicrobial resistance (AMR) is a growing public health threat, and we currently lack accurate measures to track and trend this resistance. We developed the antibiotic resistance index (ARI) that aggregates resistance of Gram-negative bacilli (GNB) into a single metric which can be tracked across healthcare settings and over time.

**Methods:**

Culture data were collected from adult patients who met the CDC adult sepsis event criteria across 10 Barnes-Jewish HealthCare (BJC) hospitals between January 2018 and December 2023. An antibiotic’s effective spectrum (AES) was calculated as the ratio of susceptible GNB to all identified GNB. The ARI was calculated as the sum of the AES to which the isolate was resistant. Using the 20 most common GNB and 15 most common anti-GNB antibiotics routinely tested in antibiograms, we calculated the ARI for each BJC hospital during the study years.

**Results:**

18,854 GNB cultured from 12,803 patients meeting CDC adult sepsis event criteria were included. AES varied between 0.15 for ampicillin and 0.94 for amikacin. *A. calcoaceticus-baumannii* complex had the highest ARI of 6.64 (IQR 4.00–9.28). Median hospital-level ARI fluctuated between 2.12 (IQR 0.40–3.83) in 2018 to 2.20 (IQR 0.34–3.86) in 2023. The ARI trajectories over time varied by medical center.

**Conclusion:**

ARI aggregates AMR in GNB and may facilitate monitoring across locations and over time. ARI and antibiotic effective spectra redefine narrow and broad spectrum of activity and offer a starting point for antibiotic utilization metrics.

## Highlights

We developed an antimicrobial resistance scoring system based on the antibiotic effective spectrum of activity to aggregate resistance of Gram-negative bacilli into a single metric that can be tracked across healthcare settings and over time.

## Introduction

Antimicrobial resistance (AMR) is one of the most significant threats to public health, with antibiotic-resistant organisms responsible for over 3 million infections yearly in the US alone.^1,2^ The Centers for Disease Control and Prevention (CDC) and the World Health Organization (WHO) have classified several Gram-negative bacilli (GNB) as serious and urgent threats.^1–3^ Tracking AMR in GNB helps inform antibiotic utilization strategies and may decrease the unwarranted and overly broad use of antibiotics. Current methods that aggregate AMR are often overly simplistic. The most used method is the hospital-specific annual antibiogram, which presents susceptibility patterns across bacterial species and antibiotics in tabulated form. While useful at the individual hospital level, this method does not aggregate the spectrum of antimicrobial activity across GNB nor allow for broader comparisons.

At the national level, trends often focus on hospital-acquired GNB, and the CDC tracks AMR in GNB as the percent of each species that is susceptible or resistant to each antibiotic across more than 2,400 acute care hospitals nationwide.^4^ Usually only certain bacterial species and antimicrobials are highlighted. This approach may leave the public and policymakers without a clear picture of the overall status of AMR since resistance trends across classes of antibiotics and microbial species might not all be in the same direction.^5^ Multidrug resistance in GNB is defined as acquired nonsusceptibility to at least one agent in three different antimicrobial classes, giving the same importance to potent broad-spectrum antibiotics and those rarely used in practice.^6^

In this project, our goal was to develop an antibiotic resistance index (ARI) that aggregates resistance across GNB. The ARI is built on the concept of effective spectrum of activity which is defined as the coverage antibiotics have across GNB, considering both intrinsic and acquired resistance. The ARI condenses AMR in GNB into a single number that can be tracked across hospitals and over time.

## Methods

### Study population and data source

This retrospective cohort study was approved by the Washington University School of Medicine Institutional Review Board (IRB number 202108211). The need for informed consent was waived. We collected data on age, sex, comorbidities, and cultures including antimicrobial susceptibility from all adult patients who met the CDC adult sepsis event criteria^7^ and were admitted to any of the 10 adult acute care BJC hospitals in Missouri and Illinois between January 2018 and December 2023. The criteria include presumed infection (sent blood cultures and at least four qualifying days of antibiotics) combined with any new concomitant organ dysfunction (vasopressor use, mechanical ventilation, increased creatinine or bilirubin levels, and thrombocytopenia). Data were directly extracted from electronic health records (EHRs).

The primary objective was to create an ARI to illustrate AMR trends in GNB responsible for serious infections and thus we limited our inclusion to culture results from septic patients, as these are more likely to reflect true infections rather than colonization and have greater impact on hospitals’ antibiotic consumption. All GNB isolated from clinically relevant specimens collected from patients with sepsis, were included as these specimens triggered the initiation of antibiotics and were deemed relevant by the treating physicians. Each microbe was included once per infectious episode (positive cultures attributed to the same infection without interim negative cultures were grouped under one episode). If a patient had two separate infections (eg, community-onset urinary tract infection followed by hospital-acquired pneumonia), both were included.

We focused on the most common 20 GNB and the most common 15 anti-GNB antibiotics routinely tested and reported in BJC hospitals’ antibiograms (Tables 1 and 2). BJC hospitals follow the Clinical and Laboratory Standards Institute (CLSI) guidelines and updates on breakpoints for antimicrobial susceptibility.^8^ During the study period, new fluoroquinolone breakpoints were implemented in 2019 after internal validation. Susceptible dose-dependent isolates were labeled as susceptible. AmpC producers, such as *Enterobacter* spp., were reported as ceftriaxone-resistant. Missing susceptibilities were manually verified. If absent, general logical rules were applied based on intrinsic resistance and accepted resistance patterns; for example, all *Pseudomonas aeruginosa* isolates were assumed resistant to ceftriaxone, and resistance to cefazolin was assumed if ceftriaxone resistance was present. Otherwise, multiple imputation by chained equations (MICE) was used to fill in missing data using 20 replications, with patient (age, sex, comorbidities, severity of illness as noted by the need for mechanical ventilation and vasopressor support), culture specimen and microbe characteristics (species and available susceptibilities) as predictors. Estimates from the multiply imputed data were combined in the usual manner according to Rubin’s rules.^9^

**Table 1.** Antibiotic effective spectrum (AES) for 15 most tested antibiotics in the 10 adult BJC hospital-specific antibiograms between 2018–2023

Antibiotic	AES	Antibiotic	AES
Ampicillin	0.15	Levofloxacin	0.78
Cefazolin	0.41	Piperacillin-tazobactam	0.82
Ampicillin-sulbactam	0.53	Cefepime	0.86
Ceftriaxone	0.56	Imipenem	0.87
Trimethoprim-sulfamethoxazole	0.64	Gentamicin	0.88
Aztreonam	0.74	Meropenem	0.91
Ceftazidime	0.77	Amikacin	0.94
Ciprofloxacin	0.77		

**Table 2.** Antibiotic resistance index (ARI) for 20 most common Gram-negative bacilli (GNB) species isolated from septic patients in the 10 adult BJC hospital between 2018–2023

GNB species	Median ARI (IQR)	GNB species	Median ARI (IQR)
*C. koseri*	0.17 (0.06–0.28)	*Acinetobacter* species (not calcoaceticus-baumannii)	1.95 (1.59–2.31)
*Klebsiella* species (not pneumoniae or oxytoca)	0.20 (0.0–0.33)	*P. stuartii*	2.06 (1.17–2.95)
*K. pneumoniae*	0.28 (0.0–0.61)	*P. aeruginosa*	2.56 (1.82–3.30)
*K. oxytoca*	0.52 (0.11–0.92)	*Pseudomonas* species (not aeruginosa)	2.86 (2.37–3.36)
*P. vulgaris*	0.59 (0.55–0.63)	*Enterobacter* species (not aerogenes)	4.05 (3.92–4.18)
*P. mirabilis*	0.65 (0.0–1.67)	*S. marcescens*	4.05 (3.95–4.15)
*P. rettgeri*	0.71 (0.47–0.94)	*C. freundii*	4.10 (3.97–4.23)
*E. coli*	0.71 (0.0–1.81)	*E. aerogenes*	4.10 (3.99–4.22)
*A. faecalis*	0.80 (0.0–1.55)	*Achromobacter* species	5.02 (4.16–5.87)
*M. morganii*	1.12 (0.11–2.13)	*A. calcoaceticus-baumannii* complex	6.64 (4.00–9.28)

17.5% infectious episodes had polymicrobial cultures with 9.8% with multiple GNB. 3.6% patients had 2 or more infectious episodes with the same pathogen separated by negative cultures (antimicrobial susceptibility may have differed).

Binary and categorical variables are expressed as percentages, and continuous variables are reported as medians (interquartile ranges).

### Analytic plan

#### Antibiotic effective spectrum (AES)

The initial step involved calculating the relative coverage of the 15 antibiotics, which was expressed as the ratio of susceptible GNB to all identified GNB. An AES value of 1 implies that all GNB cultured were susceptible to that antibiotic. Conversely, if none of the GNB were susceptible to an antibiotic, that antibiotic’s weight would be 0, as it would not provide any coverage when used as treatment. Since both intrinsic and acquired resistance determine treatment options, both were included in the analysis. Similarly to antibiograms, we calculated each antibiotic’s effective spectrum annually. Thus, the effective spectrum consolidates the information typically displayed across the antibiotic columns in antibiograms.

#### Antibiotic resistance index (ARI)

**Figure 1.**
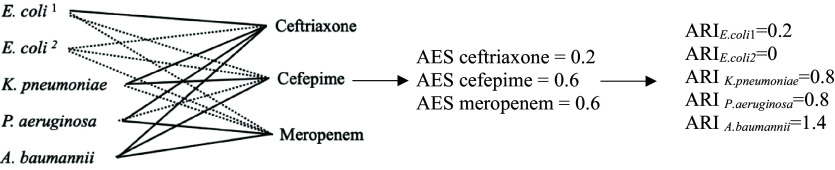
Calculation of antibiotic effective spectra (AES) and antibiotic resistance index (ARI) for a hypothetical hospital with 5 Gram-negative bacilli and 3 antibiotics. AES is the number of susceptible GNB (dashed lines) / number of all GNB (5) ARI for each microbe is the sum of all AES that the microbe is resistant to (solid lines). ARI can be averaged at unit and hospital level. See also supplementary calculations for comparisons between 2 theoretical hospitals.

For each GNB, ARI was calculated as the sum of the effective spectrum of antibiotics to which the isolate is resistant. For instance, if an *Escherichia coli* isolate is resistant to ampicillin, trimethoprim-sulfamethoxazole, and ciprofloxacin, the ARI is determined by summing the AES of these three antibiotics (Figure 1 and see also Supplementary data for examples of ARI calculation). The ARI can range from 0, indicating a pan-susceptible isolate, to the combined total of the effective spectrum of 15 antibiotics for a pan-resistant isolate. Emerging resistance to a broad(er) spectrum antibiotic with a higher AES will have a bigger impact on the microbe’s ARI than resistance to a narrower antibiotic with a lower AES.

#### ARI trends

ARI was aggregated at hospital level per year by computing the average ARI divided by the total number GNB cultured at each hospital. We report the distribution of hospital-level ARI and arbitrarily consider the 75^th^ percentile as the cutoff defining resistant GNB therefore labeling the GNB with the highest quartile ARI as resistant. We compare this cutoff to the currently used BJC definition of resistance: GNB non-susceptible to at least 3 of the following: cefepime OR ceftazidime, any carbapenem, any fluroquinolone, any anti-pseudomonal penicillin/ beta-lactamase combination (or piperacillin only if tested). *Pseudomonas* and *Acinetobacter* spp. non-susceptible to any carbapenem are also labeled as resistant GNB. Sensitivity analyses included *Stenotrophomonas maltophilia* and the 70^th^ and 80^th^ percentile for resistance cutoffs. Establishing a mathematical definition for resistant GNB that would trigger contact precautions might be useful clinically since contact precautions may reduce the spread of resistant GNB but may also be associated with reduced care and adverse patient outcomes.^10,11^ As primary end point, the pairwise differences in ARI scores in 2023 between the same five hospitals was tested via Mann–Whitney tests with a Bonferroni correction for multiple testing.

We compared hospital-specific ARI trajectories via a log-transformed linear mixed effects model with random intercept and slope effects for hospital and year, weighted by the number of cultures per hospital/year. Model fit was assessed using Akaike Information Criterion (AIC), Bayesian Information Criterion (BIC), and visual inspection of residual plots to validate model assumptions. Due to it being the largest in terms of bedsize, hospital 3 was taken as the reference group in models comparing hospital trajectories.

As more isolates become resistant to an antibiotic, the AES will decrease to a certain threshold where ARI will decrease. This threshold informs when the AES should be recalculated. We simulated a group of 100 GNB and considered only 1 antibiotic. We constructed a mathematical parabola calculating the ARI with increasing rates of resistance and thus decreasing AES.

Analyses were performed using the tidyverse, nlme, mice, circlize, and chorddiag packages in R version 4.4.0 (R Foundation for Statistical Computing, Vienna, Austria).

## Results

A total of 37,469 patients met the CDC adult sepsis event criteria over the study period. 18,854 GNB were cultured from clinical specimens in 12,803 distinct patients and included in the analyses. Most common species were *E. coli* (3646, 19.3%) cultured from urinary specimens (50.1%), *P. aeruginosa* (1855, 9.8%) predominantly from respiratory specimens (36.1%) and *Klebsiella* species (2064, 10.9%) from urine (39.0%) and blood cultures (31.1%). Our analyses did not include 1,339 (7.1%) isolates that were not part of the 20 most common species such as *S. maltophilia* and *Shigella*. *E. coli* isolates were commonly resistant to ampicillin (54.8%) while *P. aeruginosa* was resistant to meropenem in 19.9% of cultures. Resistance to meropenem and cefepime was highest in *Acinetobacter calcoaceticus—baumannii* complex (69.7% to meropenem and 49.7% to cefepime), followed by *P. aeruginosa* (12.6% resistant to cefepime).

Missingness rates were 1.0% for meropenem susceptibility testing, 4.1% for cefepime, and 2.4% for ceftriaxone. After applying the deterministic rules, and following multiple imputation, missingness rates decreased to 0.07%, 0.06% and 0.0%, respectively, due to incomplete matching between complete and incomplete cases. All available antibiotic susceptibility results were included in the analyses in incomplete cases.

**Figure 2.**
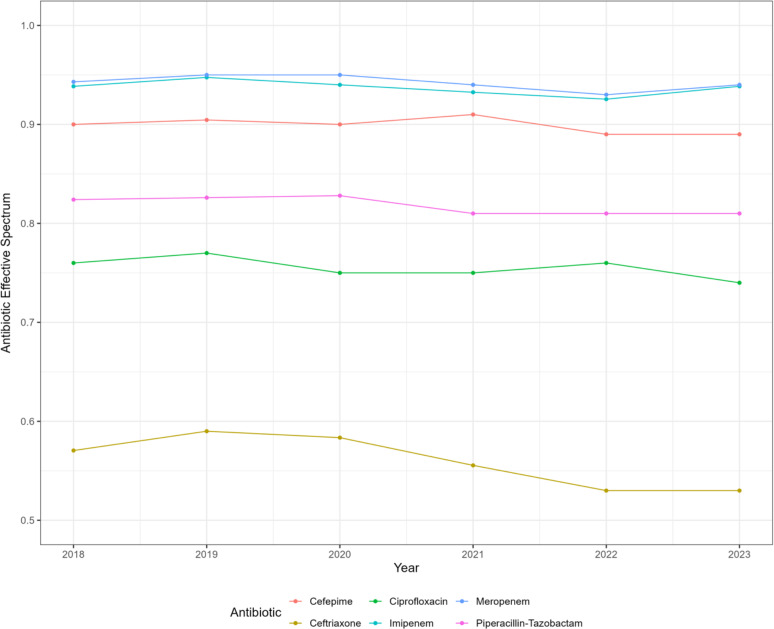
Antibiotic effective spectra (AES) for the most used antibiotics in BJC hospitals across the study years 2018–2023. All antibiotic effective spectra are shown in Supplementary Fig. 1. AES was calculated as the fraction of susceptible GNB to all GNB cultured from septic patients during each study year. AES will take values between 0 (all GNB are resistant) and 1 (all GNB are susceptible).

AES fluctuated between 0.15 for ampicillin, 0.56 for ceftriaxone and 0.82 for piperacillin-tazobactam (Table 1). Amikacin had the highest effective spectrum at 0.94. Over the 6-year study period, AES slightly decreased for all antibiotics: from 0.57 to 0.53 (7.01%) for ceftriaxone and by 1.05% from 0.95 to 0.94 for meropenem (Figure 2 and Supplementary Fig. 1 for all antibiotics included). Median ARI across GNB species varied between 0.17 (IQR 0.06–0.28) for *Citrobacter koseri* to 2.56 (IQR 1.82–3.30) for *P. aeruginosa* and 6.64 (IQR 4.00–9.28) for *A. calcoaceticus-baumannii* complex (Table 2).

**Figure 3.**
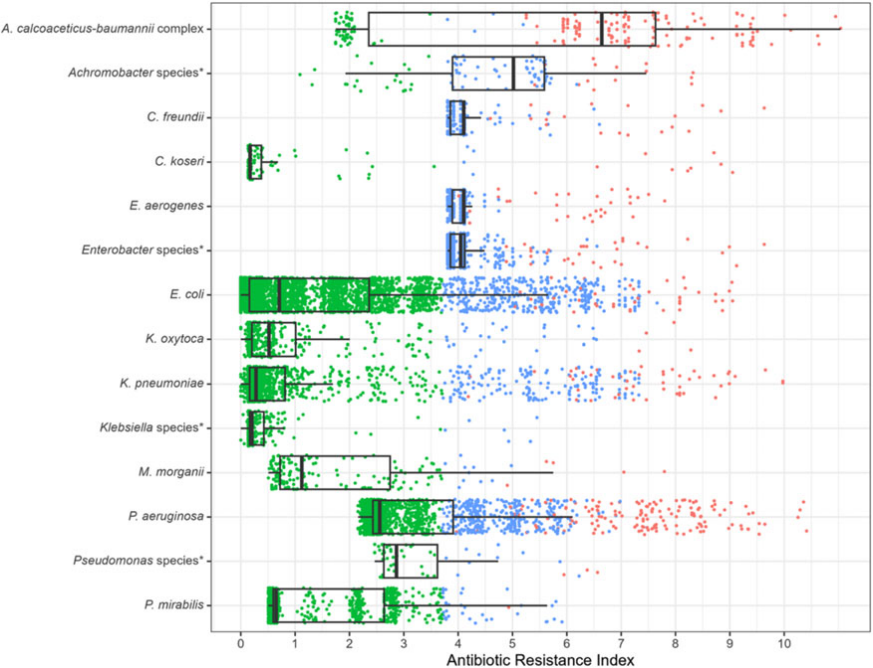
Resistant GNB definitions: BJC local definition and the 75^th^ percentile antibiotic resistance index cutoff per species of Gram- negative bacilli. BJC definition: GNB non-susceptible to at least 3 of the following: cefepime OR ceftazidime, any carbapenem (meropenem, imipenem, ertapenem, doripenem), any fluroquinolone (ciprofloxacin, moxifloxacin, levofloxacin), any anti-pseudomonal penicillin/ beta-lactamase combination (eg, piperacillin/ tazobactam) or piperacillin only if tested. *Pseudomonas* and *Acinetobacter* spp. non-susceptible to any carbapenem are also labeled as ARO. Red labels the isolates resistant by both GNB definitions (4.8%), green labels the isolates susceptible by both definitions (75.4%), and blue labels the isolates resistant by ARI 75^th^ cutoff criterion and susceptible by the BJC definition (19.8%).

The 75^th^ percentile ARI cutoff used to define resistant GNB was 3.81. There was an overlap in the distribution of resistant GNB based on the BJC definition and the 75^th^ percentile cutoff: 4.8% of isolates were classified as resistant by both definitions, while 19.8% (2010 isolates) did not meet both definitions (ie, they were labeled as susceptible by the BJC definition but resistant by the 75^th^ percentile ARI cutoff). All others were labeled as susceptible by both definitions. *E. coli, K. pneumoniae,* and *P. aeruginosa* preferentially met the ARI 75^th^ percentile cutoff but not the BJC definition (Figure 3). Lowering the ARI cutoff to the 70^th^ percentile increased the proportion of discordant isolates to 23.7% and raising the cutoff to 80^th^ percentile lowered the proportion of discordant isolated to 14.8%. Using a hospital-specific cutoff was very similar to using the system-wide calculations (Supplementary Fig. 2).

**Figure 4.**
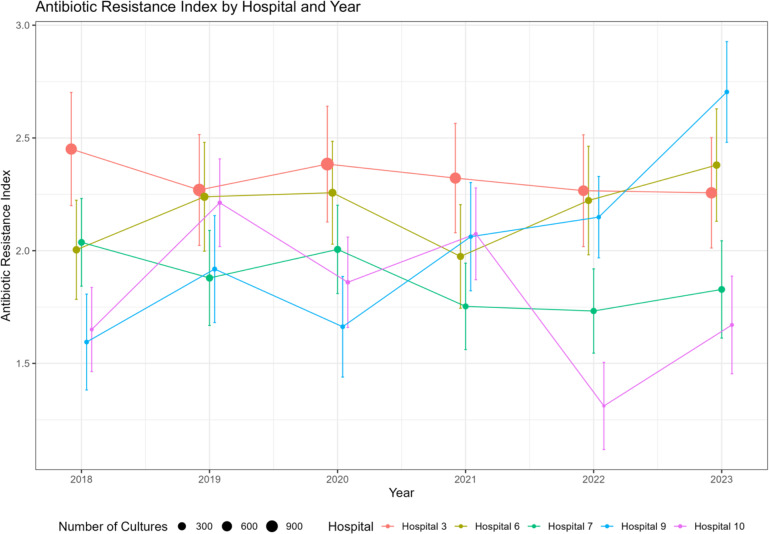
Trends in the antibiotic resistance index for 5 acute care BJC hospitals (described below) during the study years. Point-wise confidence intervals calculated using Rubin’s rules. All hospitals are shown in Supplementary Fig. 3. The antibiotic effective spectrum (AES) was calculated as the fraction of susceptible GNB to all GNB cultured from septic patients during each study year. AES will take values between 0 (all GNB are resistant) and 1 (all GNB are susceptible). ARI was calculated as the sum of the effective spectrum of antibiotics to which the isolate is resistant. For instance, if an *Escherichia coli* isolate is resistant to ampicillin, trimethoprim-sulfamethoxazole, and ciprofloxacin, the ARI is determined by summing the effective spectrum of these three antibiotics. ARI may take values between 0 (all microbes are pan-susceptible) and 10.63 (all microbes are pan-resistant to the antibiotics in Table 1). See also Supplementary data for examples of ARI calculation. Description of the 5 tables included in Fig. 4.

ARI trends during the 6 study years varied across the BJC hospitals with some hospitals experiencing increasing ARI, others relatively stable ARI and some decreases (Figure 4 for 5 representative hospitals, Supplementary Fig. 3 for all BJC hospitals). Hospital 9 had a steady increase in ARI. Much of the increase between 2022 and 2023 can be attributed to an increase in the number of microbes resistant to cefepime. From 2018 to 2022, only 10% of microbes from hospital 9 were resistant to cefepime. However, in 2023, this percentage increased to 26%. Since cefepime has a relatively large AES, resistance to this antibiotic greatly increases the ARI. In 2023, hospital 9 had significantly higher ARI than hospitals 6 and 10 (*P* < .001, *P* < .001). Hospital 9 had a significantly different ARI trajectory across the study period compared to the reference hospital, hospital 3 (*β* = 0.106, standard error = 0.032, *P* = .005). When including *S. maltophilia*, most hospitals had higher ARI, in particular hospital 3, which is the referral cancer and transplant center for the region (Supplementary Fig. 4).

The simulations showed the ARI is highest when 50% of microbes are resistant to an antibiotic (AES 0.5, ARI 0.25, Supplementary Table 1). As the number of resistant isolates crosses the 50% threshold, the AES decreases leading to lower ARI despite higher numbers of resistant isolates. To allow for accurate ARI calculations, AES should be updated yearly when close to the 50% threshold.

## Discussion

In this multicenter retrospective study, we aggregated AMR in GNB and tracked the ARI, a composite measure, across 10 adult hospitals in the Midwest. The ARI is based on the effective spectrum of antibiotics, calculated as the number of GNB susceptible to a specific antibiotic out of the total number of GNB at that institution. This approach redefines narrow and broad spectrum based on the true remaining antibiotic coverage. ARI can be calculated for each microbe and aggregated at unit and hospital levels facilitating AMR monitoring across hospitals and over time. We included both intrinsic and acquired resistance, as both determine antibiotic choices.

Communicating the importance of AMR has been challenging due to the multitude of species and antibiotics involved. Starting in 2025, the CDC will release estimates for at least 19 AMR threats and update the U.S. burden of AMR by pathogen.^1^ While this is informative, it may not capture the full picture of resistance, as some trends may increase while others remain stable. Even slight changes may have a significant impact over a longer period and our results show down-trending spectrum of activity for the antibiotics considered. By aggregating resistance to the 15 most-used anti-GNB antibiotics, we provide a composite index that may help communication about the overall trajectory of AMR in GNB responsible for serious infections. Clinically relevant changes in resistance, such as carbapenemase-producing isolates, will result in significant ARI increases due to the high AES assigned to carbapenems. Similar to antibiograms, ARI can help identify which antibiotics contribute most to resistance changes over time (see case study of hospital 9) and it will require updating, especially as resistance approaches 50%. ARI and its AES components can contribute to developing institution-specific guidelines based on local resistance patterns. As a dynamic measure, ARI can incorporate new antibiotics as they enter routine testing and use and it can also exclude antibiotics as they phase out of clinical care. Part of future external validation efforts, a representative group of antibiotics, tested in all hospitals’ antibiograms should be defined.

Previous efforts to aggregate AMR include the drug resistance index developed by Laxminarayan et al.^12^ This index, which is similar to composite economic indices, combines the percentage of resistant GNB to certain antibiotics with the usage rates of those antibiotics. Lower values are preferred. While it can show changes in AMR and prescribing practices over time, this approach may inadvertently encourage the unnecessary use of broad-spectrum antibiotics since overly broad antibiotics will decrease the use of the initial antibiotic.^13–15^ In the future, the AES included in the ARI could be used to select the narrowest spectrum antibiotics (ie, lowest effective spectrum) and measure antibiotic utilization more precisely. Traditionally, antibiotic utilization in hospitalized patients is assessed by volume, commonly using days of therapy per 1,000 patient-days. Studies by Gerber et al., Sullivan et al., Lahart et al., and Kakiuchi et al. evaluated antibiotic spectrum (the microbial classes covered by each antibiotic) indices for antimicrobial stewardship, focusing on reducing the antibiotic spectrum in various settings and demonstrating that spectrum adds a new dimension to antibiotic use monitoring.^16–19^ These indices combine coverage for Gram-positive, Gram-negative, atypical, and anaerobic bacteria, resulting in higher scores for some antibiotics regardless of specific resistance patterns in GNB. Our study focuses on GNB and constructs the AES and ARI based on the retained coverage across GNB, providing a background for resistance that may justify or disprove certain antibiotic utilization practices.

This study has several limitations. Even though our study is based on data from 10 hospitals, it may not be representative of culture data in other hospitals. We included only culture data from septic patients, potentially excluding milder infections that do not cause organ dysfunction. We wanted to focus on serious infections that warrant antibiotic treatment and avoid potential colonizers and cultures without clinical consequences. We focused on the most common GNB and did not include less tested strains such as *S. maltophilia*. We started with the premise that higher resistance requires broader antibiotics, which is not always the case with *S. maltophilia*. Future iterations may test whether the ARI changes when all cultures, not just from septic patients, are included and may focus on transmissible resistance by excluding intrinsic resistance for infection prevention and control purposes. Having broader inclusion criteria similar to antibiograms may allow broader generalizability to hospitals with limited resources. Most hospitals track sepsis performance metrics which could be used for AES and ARI calculations. Our method aims to rank antibiotics based on their effective coverage and to sum up both intrinsic and extrinsic resistance across GNB at the hospital level. Although, for most antibiotics, a higher AES translates into broader coverage and potentially larger clinical use, our method does not imply clinical utility. For instance, aminoglycosides have the highest AES without having the highest clinical utility in the treatment of GNB. Systematic missingness for testing certain antibiotics like ertapenem and moxifloxacin was noted in BJC hospitals. Changing CLSI breakpoints may affect the classification of resistant and susceptible labels. During our study, only the breakpoints for fluoroquinolones were modified. However, as shown in Figure 2, the AES for ciprofloxacin continued to decrease in 2023 compared to 2020. Future studies could expand the number of antibiotics included, reflecting more comprehensive testing practices. AES should also be updated as coverage changes significantly, maintaining the ARI’s relevance in tracking resistance similar to yearly antibiogram updates. We have provided a case discussion on how AES and ARI may be used in practice.

In conclusion, the ARI, based on the AES, presents an aggregated measure of AMR in GNB responsible for serious infections. It enables tracking of resistance trends across hospitals and over time and can incorporate new antibiotics as they become available. AES redefines narrow and broad spectrum of activity and offers a starting point for antibiotic utilization metrics. Public health initiatives aimed at decreasing AMR may be assessed by measuring ARI in the future.

## Supporting information

10.1017/ash.2025.10275.sm001 Vazquez Guillamet et al. supplementary material 1 Vazquez Guillamet et al. supplementary material

10.1017/ash.2025.10275.sm002 Vazquez Guillamet et al. supplementary material 2 Vazquez Guillamet et al. supplementary material

10.1017/ash.2025.10275.sm003 Vazquez Guillamet et al. supplementary material 3 Vazquez Guillamet et al. supplementary material

10.1017/ash.2025.10275.sm004 Vazquez Guillamet et al. supplementary material 4 Vazquez Guillamet et al. supplementary material

## Supplementary material

The Supplementary material for this article can be found at https://doi.org/10.1017/ash.2025.10275.

## Data sharing

De-identified data will be available via Washington University’s Becker Medical library. Analytic code is available at https://github.com/afbewley/abx_resistance_index.
