# Supplementary figures and images for: Effective spectrum-based antibiotic resistance index for monitoring resistance in Gram-negative bacilli
Source: Antimicrob Steward Healthc Epidemiol. 2026 Mar 27;6(1):e74. doi: 10.1017/ash.2025.10275 (PMC13040296; doi:10.1017/ash.2025.10275)

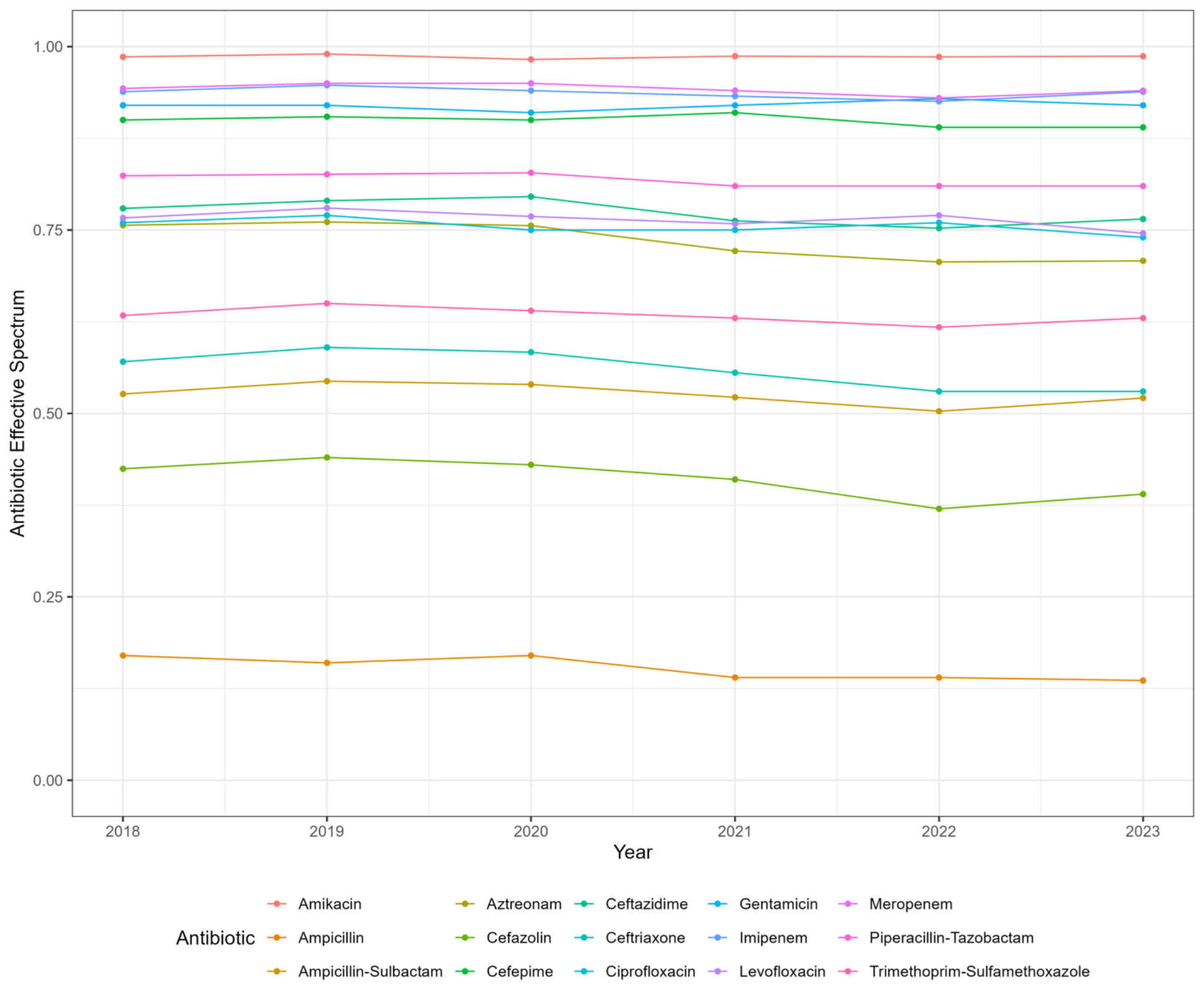

Supplement: Vazquez Guillamet et al. supplementary material 1 — Vazquez Guillamet et al. supplementary material [file S2732494X25102751sup001.tiff]

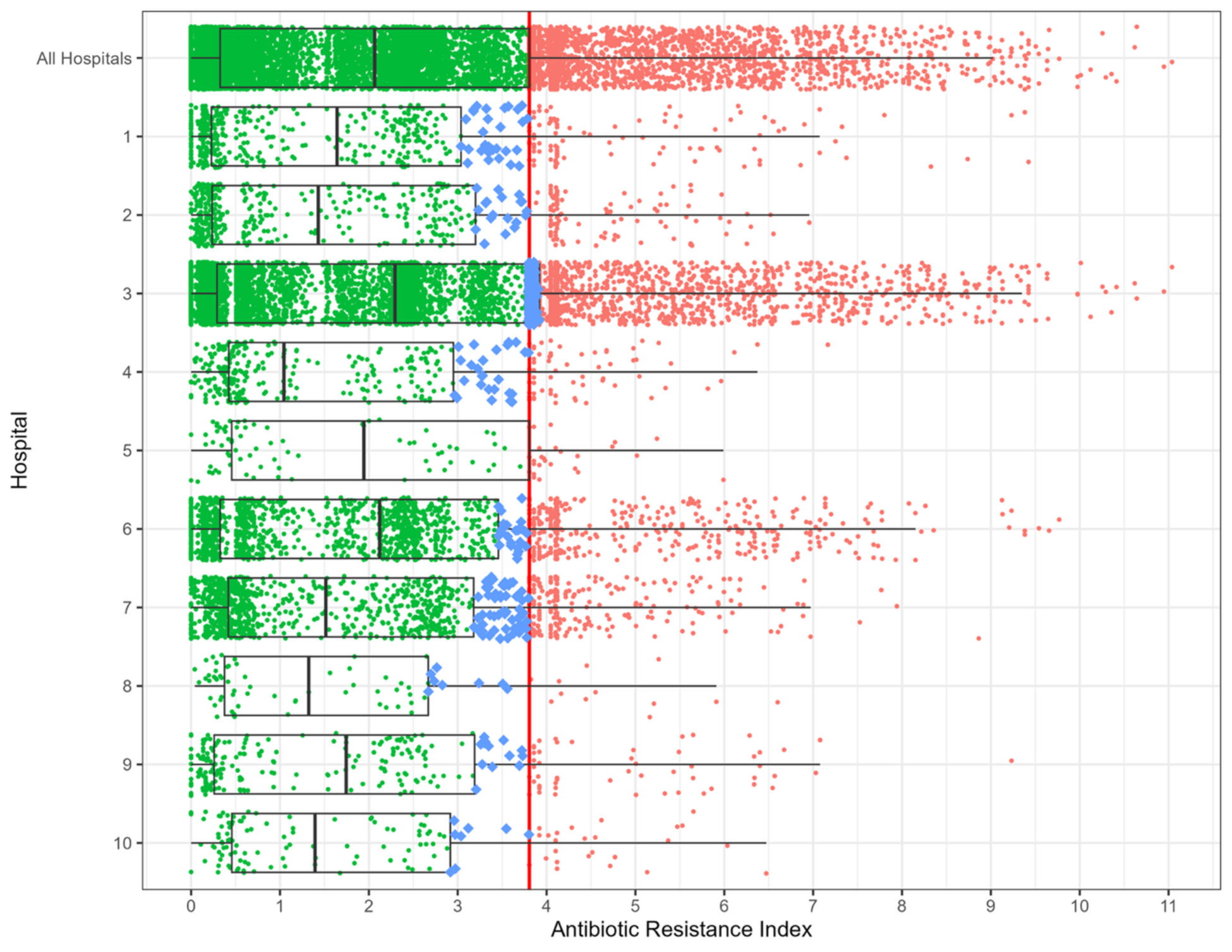

Supplement: Vazquez Guillamet et al. supplementary material 2 — Vazquez Guillamet et al. supplementary material [file S2732494X25102751sup002.tiff]

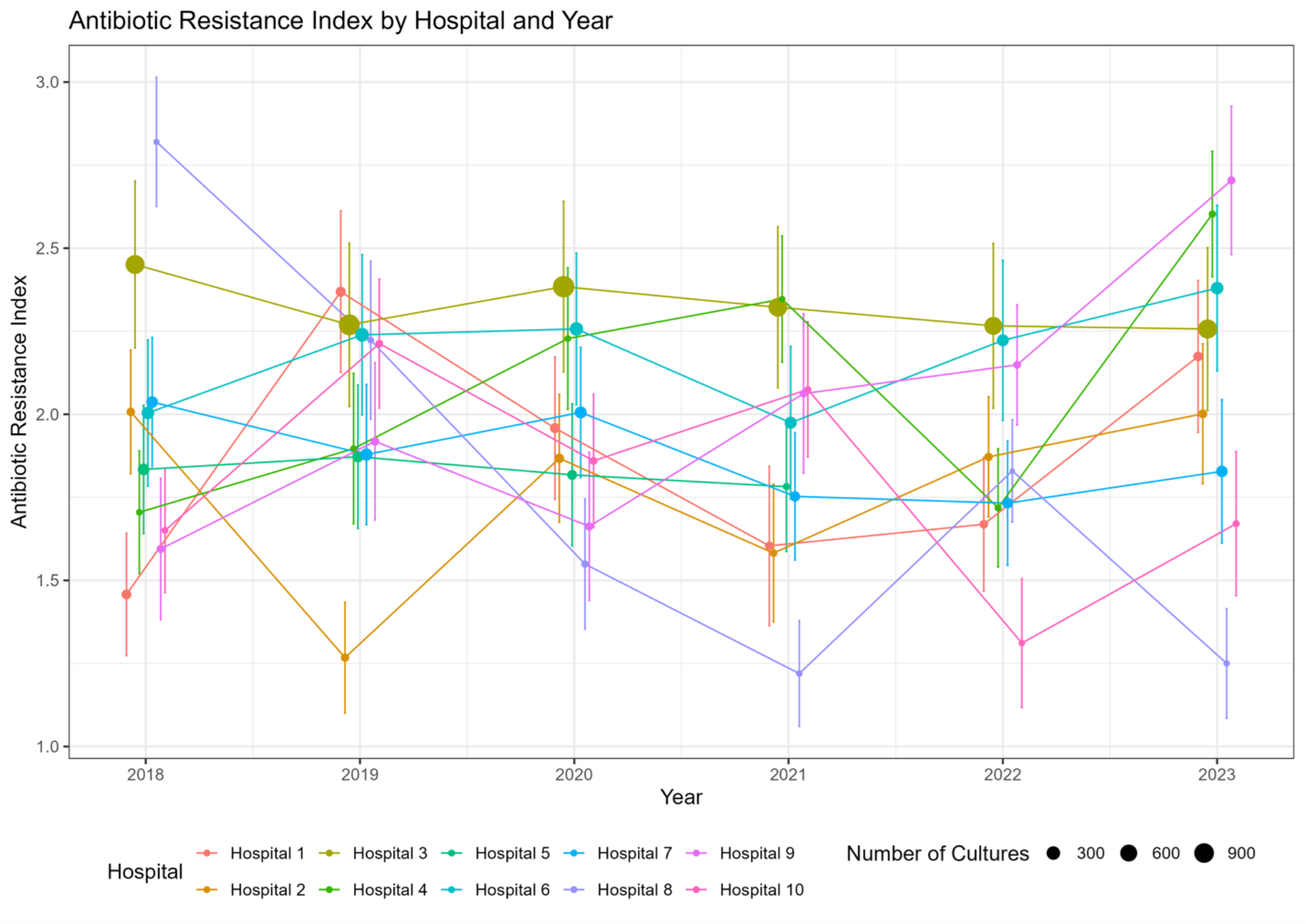

Supplement: Vazquez Guillamet et al. supplementary material 3 — Vazquez Guillamet et al. supplementary material [file S2732494X25102751sup003.tiff]

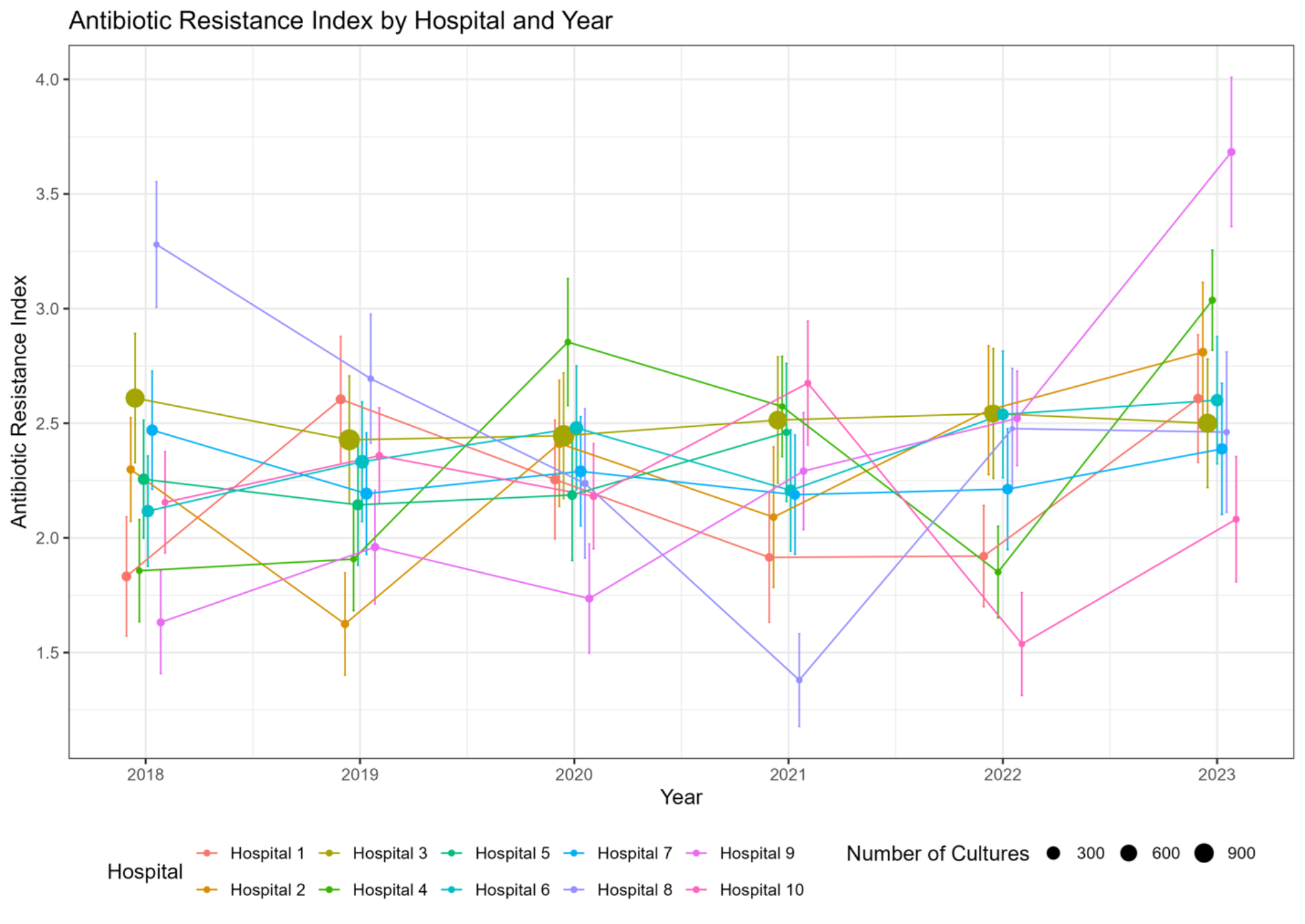

Supplement: Vazquez Guillamet et al. supplementary material 4 — Vazquez Guillamet et al. supplementary material [file S2732494X25102751sup004.tiff]
